# No clinically meaningful differences between PCL preservation and sacrifice in medial pivot total knee arthroplasty: A systematic review and meta‐analysis

**DOI:** 10.1002/jeo2.70559

**Published:** 2025-11-14

**Authors:** Amir Mohsen Khorrami, Mohammad Rastegar, Mehdi Mohammadpour, Mohammad Ayati Firoozabadi, Seyed Mohammad Javad Mortazavi

**Affiliations:** ^1^ Bone and Joint Reconstruction Research Center, Department of Orthopedics, School of Medicine Iran University of Medical Sciences Tehran Iran; ^2^ Joint Reconstruction Research Center Tehran University of Medical Sciences Tehran Iran; ^3^ Department of Orthopedic Surgery Imam Khomeini Hospital Complex Tehran University of Medical Sciences Tehran Iran

**Keywords:** functional outcome, medial pivot prostheses, posterior cruciate ligament preservation, systematic review, total knee arthroplasty

## Abstract

**Purpose:**

The decision to preserve or sacrifice the posterior cruciate ligament (PCL) during medial pivot total knee arthroplasty (TKA) remains controversial. This systematic review and meta‐analysis evaluated postoperative functional outcomes and complications in patients undergoing medial pivot TKA with and without PCL preservation.

**Methods:**

Eight databases were systematically searched eight databases through July 2025 following PRISMA guidelines. Studies comparing PCL preservation (cruciate‐retaining [CR]) versus sacrifice (cruciate‐sacrificing [CS]) in medial pivot TKA with minimum 2‐year follow‐up were included. Primary outcomes were functional scores and complication rates. Due to substantial heterogeneity (*I* ^2^ > 75%) in key outcomes, we emphasized qualitative synthesis over pooled estimates for affected outcomes. Statistical analysis employed random‐effects models with prediction intervals for highly heterogeneous outcomes.

**Results:**

Seven cohort studies (957 patients) were included. For outcomes with acceptable heterogeneity, quantitative pooling showed no statistically significant differences: Knee Society Score (KSS) (mean difference: 0.39, 95% confidence interval [CI]: −0.5, 1.27, *I* ^2^ = 60.4%) and Forgotten Joint Score (FJS) (mean difference: 0.77, 95% CI: −0.25, 1.79, *I* ^2^ = 23%). However, Western Ontario and McMaster Universities Osteoarthritis Index (WOMAC), Oxford Knee Score and range of motion demonstrated extremely high heterogeneity (*I* ^2^ = 89.6%–99.4%), precluding reliable pooled estimates. Qualitative synthesis of individual studies consistently showed no clinically meaningful differences between approaches, it is important to note that the extremely high heterogeneity for most functional outcomes severely limits the reliability of these conclusions and prevents definitive recommendations. Complication rates were similar between groups (9% CS vs. 6% CR, *p* = 0.57, *I* ^2^ = 21%).

**Conclusions:**

Based on low to moderate certainty evidence with significant study heterogeneity, PCL management strategy does not result in clinically meaningful differences in functional outcomes or complication rates in medial pivot TKA.

**Level of Evidence:**

Level III.

**Abbreviations**
CR: cruciate‐retaining
CS: cruciate‐sacrificing
FJS: Forgotten Joint Score
KOOS: Knee Injury and Osteoarthritis Outcome Score
KSS: Knee Society Score
OKS: Oxford Knee Score
PCL: posterior cruciate ligament
PICO: Population Intervention Comparison Outcomes
TKA: total knee arthroplasty
WOMAC: Western Ontario and McMaster Universities Osteoarthritis Index

## INTRODUCTION

Total knee arthroplasty (TKA) effectively relieves pain and improves function in patients with end‐stage osteoarthritis, with over 700,000 procedures performed annually in the United States [12, 31]. Despite significant advances in implant design and surgical techniques, replicating normal knee kinematics remains challenging [6, 27]. The medial pivot design, introduced in 2002, represents an innovative approach that creates a fixed centre of rotation medially while allowing lateral femoral rollback, more closely mimicking natural knee mechanics [5, 7].

The medial pivot prosthesis features a ball‐and‐socket configuration in the medial compartment providing stability, while the lateral compartment permits smooth rolling and gliding motion [17, 22, 25]. This design theoretically offers improved stability and more natural kinematics compared to traditional implants [21, 23]. However, the optimal management of the posterior cruciate ligament (PCL) in medial pivot TKA remains controversial.

Over the past decade, TKA has significantly improved clinical and functional outcomes, partly thanks to innovative implant designs, advanced prosthetic materials and refined surgical techniques [14, 19]. TKA aims to restore normal movement and function of the knee. However, neither traditional nor modern designs, whether they retain or replace the PCL, have successfully replicated the kinematics and stability of a healthy, nonarthritic knee.

Current evidence regarding PCL management in conventional TKA designs suggests no clear superiority of either approach, with meta‐analyses showing equivalent functional outcomes and complication rates [30]. However, the unique kinematic properties of medial pivot designs may theoretically alter the importance of PCL preservation, as the inherent stability provided by the ball‐and‐socket medial configuration could potentially compensate for PCL sacrifice [27]. Conversely, PCL preservation might provide additional proprioceptive benefits and natural femoral rollback that could enhance the already favourable kinematics of medial pivot designs.

The theoretical rationale for PCL preservation includes maintained proprioception, preserved natural femoral rollback and enhanced posterior stability [18, 26]. Conversely, PCL sacrifice may facilitate easier soft tissue balancing, provide more predictable kinematics and eliminate concerns about PCL integrity over time [24, 30]. Some argue that the medial pivot design's inherent stability may render PCL status less critical [27].

Previous meta‐analyses have examined PCL management in conventional TKA designs, but none have specifically focused on medial pivot prostheses [30]. This represents an important knowledge gap, as the unique kinematic properties of medial pivot designs may influence the clinical significance of PCL preservation decisions.

Therefore, this systematic review and meta‐analysis were conducted to evaluate whether PCL preservation affects functional outcomes and complication rates in medial pivot TKA. The primary hypothesis was that PCL preservation would not result in clinically meaningful differences in functional outcomes compared to PCL sacrifice in patients receiving medial pivot TKA.

## METHODS

### Protocol and registration

This systematic review was conducted according to PRISMA 2020 guidelines and registered with PROSPERO (CRD42025108757). The protocol was established a priori to minimize bias and ensure methodological rigour.

### Search strategy

PubMed, Medline, Cochrane Library, Scopus, Google Scholar, Web of Science and Embase were systematically searched PubMed, Medline, Cochrane Library, Scopus, Google Scholar, Web of Science and Embase from inception through July 2025. The search strategy was developed using the Population Intervention Comparison Outcomes (PICO) framework:

- **Population:** Adults undergoing TKA.
- **Intervention:** Medial pivot prostheses with PCL preservation.
- **Comparison:** Medial pivot prostheses with PCL sacrifice.
- **Outcomes:** Functional scores, complications and range of motion (ROM).

Search terms included: (‘Total Knee Replacement’ OR ‘Knee Arthroplasty’ OR ‘Total Knee Arthroplasty’) AND (‘medial pivot’ OR ‘medial rotating’ OR ‘medial‐stabilized’ OR ‘medial ball‐and‐socket’ OR ‘medial pivot implant’ OR ‘medial pivot prostheses’) AND (‘Posterior Cruciate Ligament preservation’ OR ‘posterior cruciate ligament retention’ OR ‘posterior cruciate ligament sacrifice’ OR ‘posterior cruciate ligament removal’).

### Eligibility criteria

#### Inclusion criteria

- Comparative studies evaluating medial pivot TKA with and without PCL preservation.
- Studies specifically using medial ball‐and‐socket type implants with a constrained medial compartment and mobile lateral compartment design.
- Validated functional outcome measures (Knee Society Score [KSS], Western Ontario and McMaster Universities Osteoarthritis Index [WOMAC], Oxford Knee Score [OKS], Forgotten Joint Score [FJS], Knee Injury and Osteoarthritis Outcome Score [KOOS]) and/or complication data.
- Minimum 2‐year follow‐up (justified as necessary for capturing late complications and functional recovery).
- Minimum 20 patients per group.
- English language publications.

#### Exclusion criteria

- Studies evaluating only conventional (nonmedial pivot) prostheses
- Studies using only medial‐stabilized designs without true medial ball‐and‐socket configuration.
- Single‐arm studies without comparison groups.
- Mixed prosthesis types without separate medial pivot analysis.
- Case reports and case series (<10 patients).
- Lack of full‐text availability.
- Reviews and meta‐analyses.

### Study selection and data extraction

Two reviewers independently screened titles, abstracts and full texts. Disagreements were resolved through discussion or third reviewer consultation. Data extraction included study characteristics, patient demographics, surgical details, functional outcomes, complications and follow‐up duration. Authors were contacted for missing data when possible.

### Quality assessment

Study quality was assessed using the Newcastle‐Ottawa Scale for cohort studies [26], evaluating selection, comparability and outcome domains. Studies were classified as poor (0–3 points), fair (4–6 points) or good (7–9 points). Evidence certainty was evaluated using the Grading of Recommendations Assessment, Development and Evaluation (GRADE) approach [15], considering study design limitations, heterogeneity, imprecision, indirectness and publication bias.

### Statistical analysis

Statistical analysis was performed using Stata version 17. Given expected clinical heterogeneity, random‐effects models were used throughout. For outcomes with substantial heterogeneity (*I* ^2^ > 75%), we calculated prediction intervals and prioritized qualitative synthesis over pooled estimates. Heterogeneity was assessed using Cochran's *Q* test and *I* ^2^ statistics, interpreted as: 0%–40% (not important), 30%–60% (moderate), 50%–90% (substantial), 75%–100% (considerable). For highly heterogeneous outcomes (*I* ^2^ > 75%), we emphasized individual study results and provided cautious interpretation of pooled estimates, acknowledging their limited reliability. Meta‐regression was performed to explore sources of heterogeneity, examining factors including sample size, study quality, mean age, follow‐up duration and geographic region. Publication bias was assessed using Egger's test and funnel plots when ≥10 studies were available.

#### Power analysis

Post hoc power calculations were performed for primary outcomes using observed effect sizes and confidence intervals.

### Clinical interpretation

When interpreting between‐group differences, we applied established minimal clinically important differences (MCIDs) as reference values for clinical significance, while acknowledging that originally developed to assess individual patient improvement rather than between‐group comparisons: MCIDs were KSS (5.3 points) [11], WOMAC (10.2 points) [1], OKS (4.5 points) [3], FJS (6.4 points) [4] and ROM (5.7°) [28]. Between‐group differences substantially below these reference values were considered unlikely to represent clinically meaningful differences.

## RESULTS

### Study selection and characteristics

**Figure 1:**
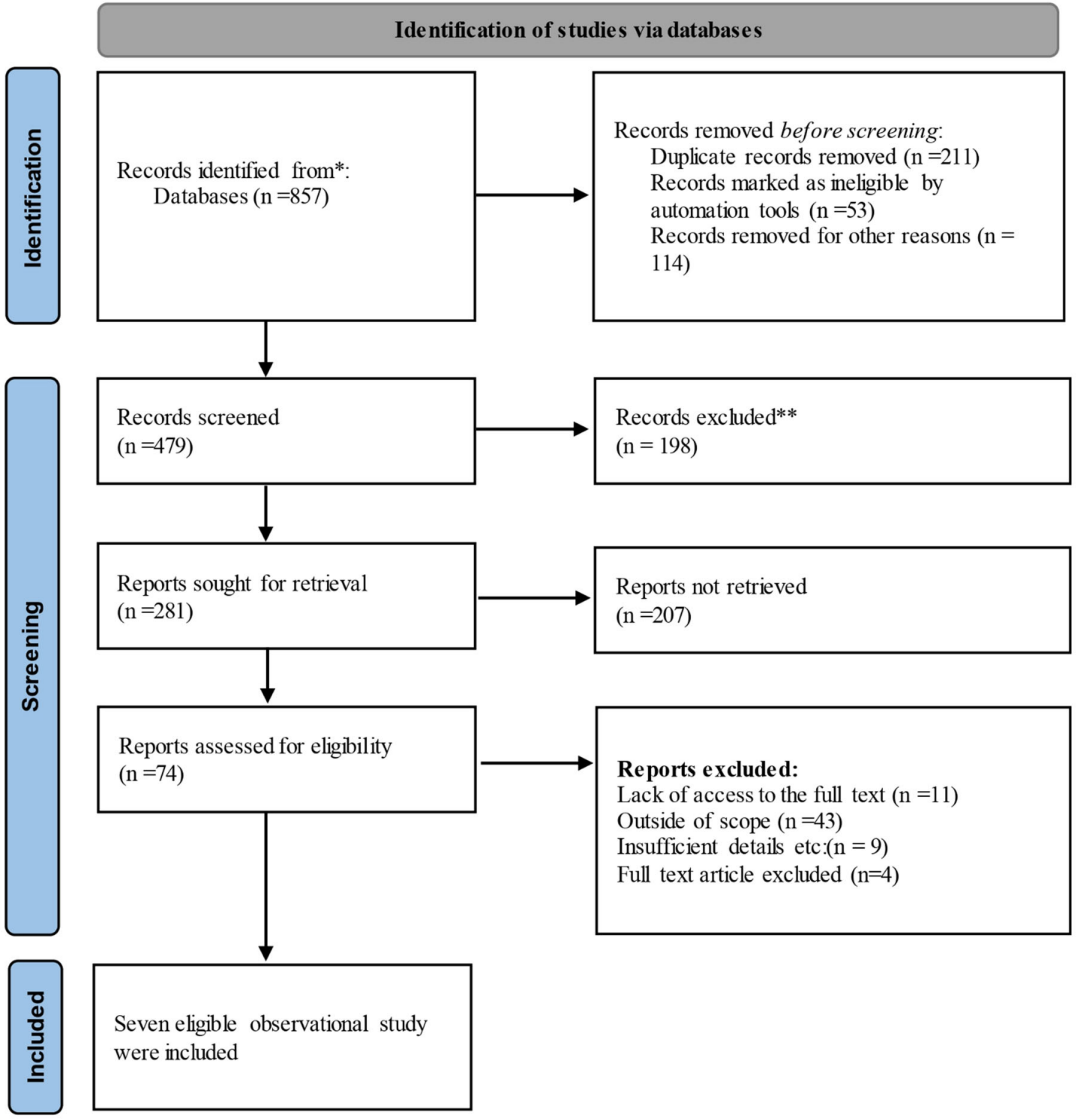
Flowchart page of studies based on PRISMA 2020. *Databases searched: PubMed, Medline, Cochrane Library, Scopus, Google Scholar, Web of Science, and Embase (from inception through July 2025). **Records excluded during title and abstract screening based on predetermined exclusion criteria.

The systematic search identified 857 articles. After removing 211 duplicates, 646 titles and abstracts were screened. Following full‐text review of 74 articles, seven cohort studies met the inclusion criteria (Figure 1). Excluded studies (*n* = 67) were rejected primarily due to: mixed prosthesis types without separate analysis (*n* = 23), single‐arm design (*n* = 18), insufficient follow‐up (*n* = 12), conventional prostheses only (*n* = 8) and other reasons (*n* = 6).

Seven cohort studies [2, 8, 9, 13, 16, 20, 29] including 957 patients were analysed. Six studies were prospective cohorts and one was retrospective. The weighted mean age was 71.6 years (range: 67.5–78.0), mean body mass index (BMI) was 26.4 ± 2.1 kg/m^2^ and 72% were female. Geographic distribution included four Asian studies (57%), two European studies (29%) and one Australian study (14%).

Study quality assessment revealed three good‐quality studies and four fair‐quality studies based on Newcastle‐Ottawa Scale scores. The mean follow‐up was 6.3 ± 3.3 years (range: 2.0–10.7 years). Most studies did not adequately describe the decision‐making process for PCL preservation versus sacrifice, representing a potential source of selection bias. Patient characteristics are summarized in Table 1.

**Table 1:** Characteristics of patients in studies and quality of included studies.

Author(s)	Country	Study design	Sample size	CR/CS	Sex (female)	BMI	Mean follow‐up (year)	Age	Surgical technique/alignment	PCL management decision criteria	Quality of studies	Certainty of evidence
Bae et al. (2011) [2]	South Korea	Prospective cohort	119	59/60	112	26.2	2	67.5	Mechanical alignment	Not specified	Fair	High
Brinkman et al. (2014) [8]	Australia	Prospective cohort	50	27/23	22	NA	9.6	69	Mechanical alignment	Not specified	Fair	Low
Macheras et al. (2017) [20]	Greece	Prospective cohort	325	176/149	215	28.9	6.2	78	Mechanical alignment	Not specified	Good	High
Ueyama et al. (2021) [29]	Japan	Prospective cohort	333	76/257	308	23.4	10.2	75.2	Mechanical alignment	Not specified	Good	High
Budhiparama et al. [9]	Indonesia	Prospective cohort	66	33/33	24	25.8	2.81	70.1	Kinematic alignment	Randomized allocation	Fair	Moderate
Hu et al. (2023) [16]	China	Prospective cohort	376	84/292	NA	27.9	10.7	68.2	Mechanical alignment	Not specified	Good	Moderate
Giustra et al. (2023) [13]	Italy	Retrospective cohorts	64	35/29	37	25.9	2.6	73.4	Kinematic alignment	Not specified	Fair	Moderate

*Note*: All studies used the Advance Medial Pivot (Wright Medical) implant model.

Abbreviations: BMI, body mass index; CR, cruciate‐retaining; CS, cruciate‐sacrificing; NA, not applicable; PCL, posterior cruciate ligament.

### Functional outcomes

Given the extremely high heterogeneity observed in most functional outcomes, which severely limits the reliability of pooled estimates, results emphasizing qualitative synthesis over quantitative pooling where appropriate are presented.

### Outcomes with acceptable heterogeneity for pooling

#### Knee Society Score (KSS)

**Figure 2:**
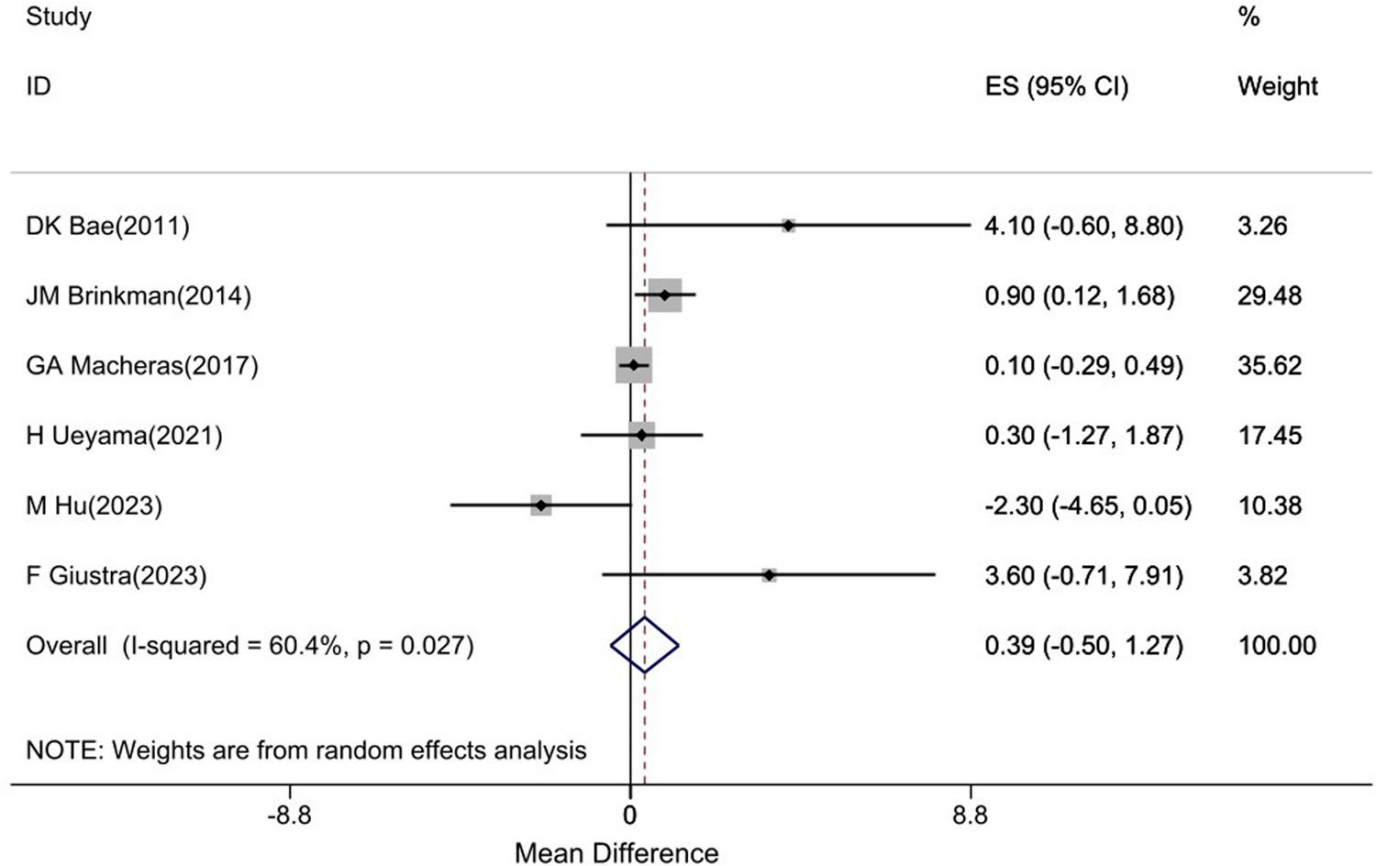
Difference in mean postoperative KSS score between CR and CS groups (positive values favour CR). CI, confidence interval; CR, cruciate‐retaining; CS, cruciate‐sacrificing; ES, effect size; KSS, Knee Society Score. *Note*: Weights are from random effects analysis.

Six studies [2, 8, 13, 16, 20, 29] reported KSS scores with moderate heterogeneity (*I* ^2^ = 60.4%). The pooled estimate showed no statistically significant difference between cruciate‐retaining (CR) and cruciate‐sacrificing (CS) groups (mean difference: 0.39, 95% confidence interval [CI]: −0.5, 1.27). This difference is well below the MCID reference value of 5.3 points, suggesting no clinically meaningful difference between groups (Figure 2).

#### Forgotten Joint Score (FJS)

**Figure 3:**
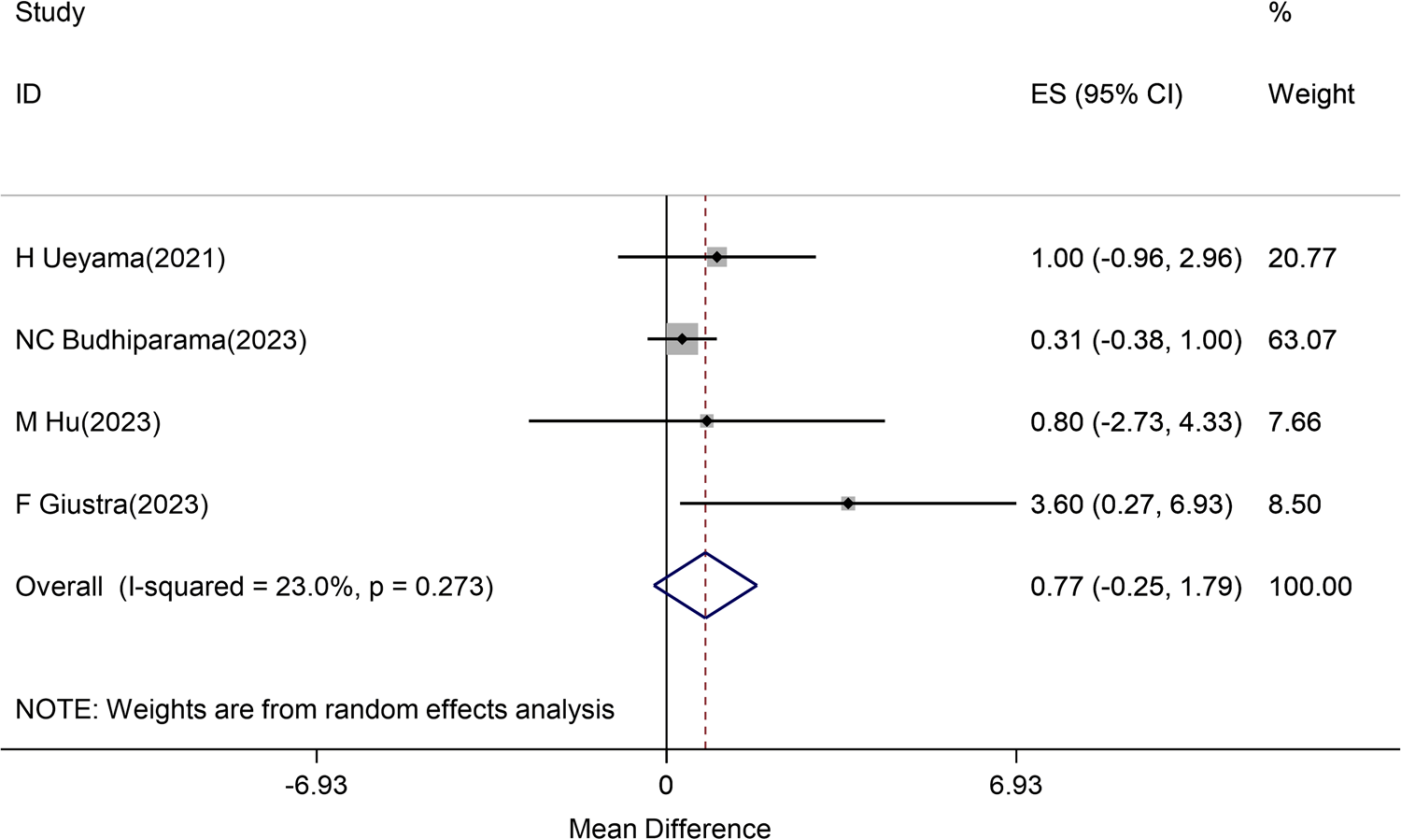
Difference in mean postoperative FJS score between CR and CS groups positive values favour CR). CI, confidence interval; CR, cruciate‐retaining; CS, cruciate‐sacrificing; ES, effect size; FJS, Forgotten Joint Score. *Note*: Weights are from random effects analysis.

Four studies [8, 13, 16, 29] reported FJS scores with low heterogeneity (*I* ^2^ = 23%). The pooled estimate showed no significant difference (mean difference: 0.77, 95% CI: −0.25, 1.79), well below the MCID of 6.4 points, well below the MCID reference value of 6.4 points (Figure 3).

### Outcomes with extremely high heterogeneity—qualitative synthesis

#### WOMAC Score

Three studies [8, 16, 20] reported WOMAC scores with extremely high heterogeneity (*I* ^2^ = 89.6%), making pooled estimates unreliable. Individual study analysis revealed:

- Brinkman et al.: No significant difference (*p* = 0.43).
- Hu et al.: Favoured CS group by 2.1 points (not clinically significant).
- Macheras et al.: Favoured CR group by 1.8 points (not clinically significant).

**Figure 4:**
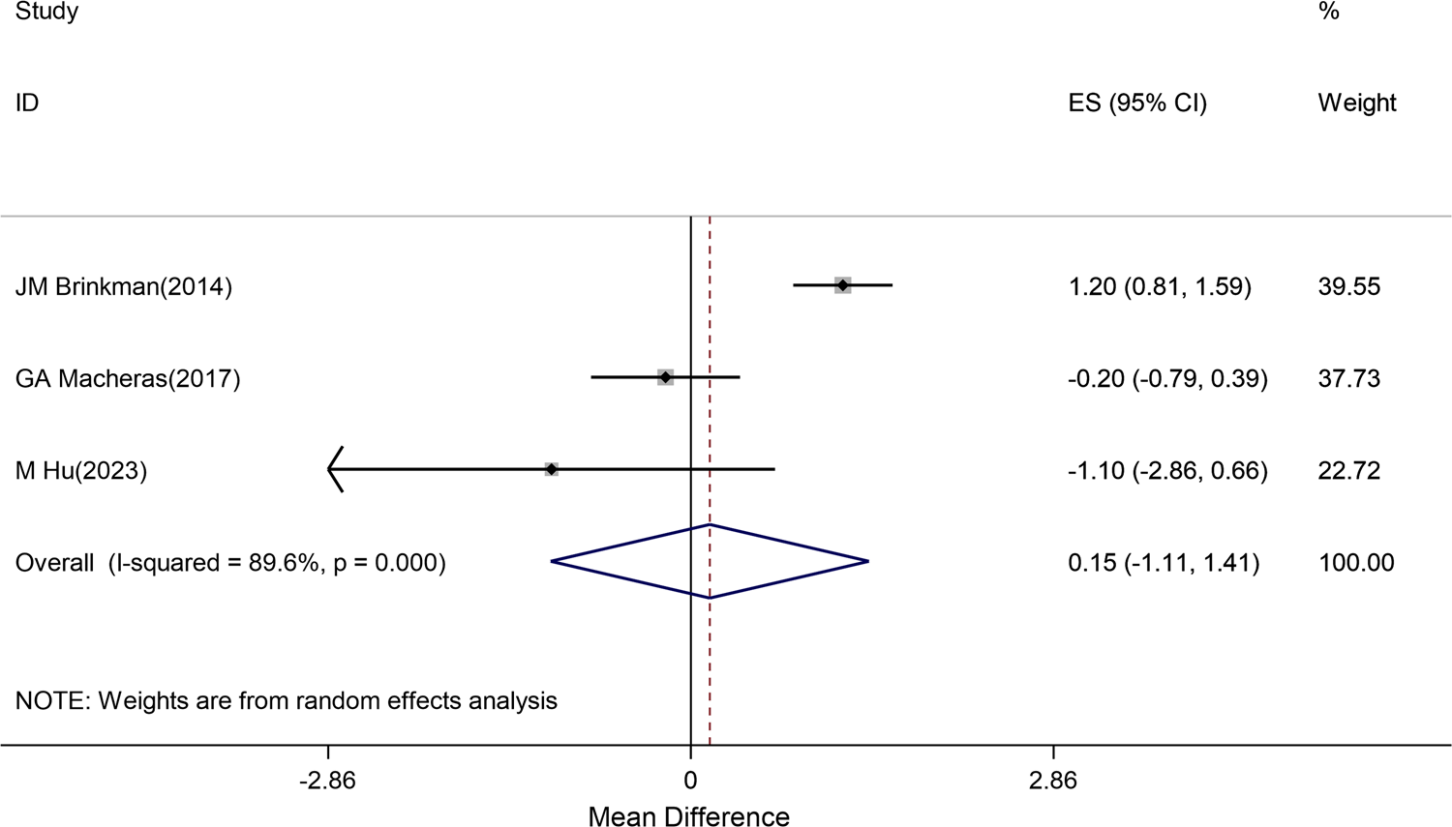
Difference in mean postoperative WOMAC score between CR and CS groups positive values favour CR). CI, confidence interval; CR, cruciate‐retaining; CS, cruciate‐sacrificing; ES, effect size. WOMAC, Western Ontario and McMaster Universities Osteoarthritis Index. *Note*: Weights are from random effects analysis.

All individual study differences were below the MCID reference value of 10.2 points. The prediction interval was extremely wide (−15.3 to 15.6), indicating substantial uncertainty in treatment effects across different settings (Figure 4).

#### Oxford Knee Score (OKS)

**Figure 5:**
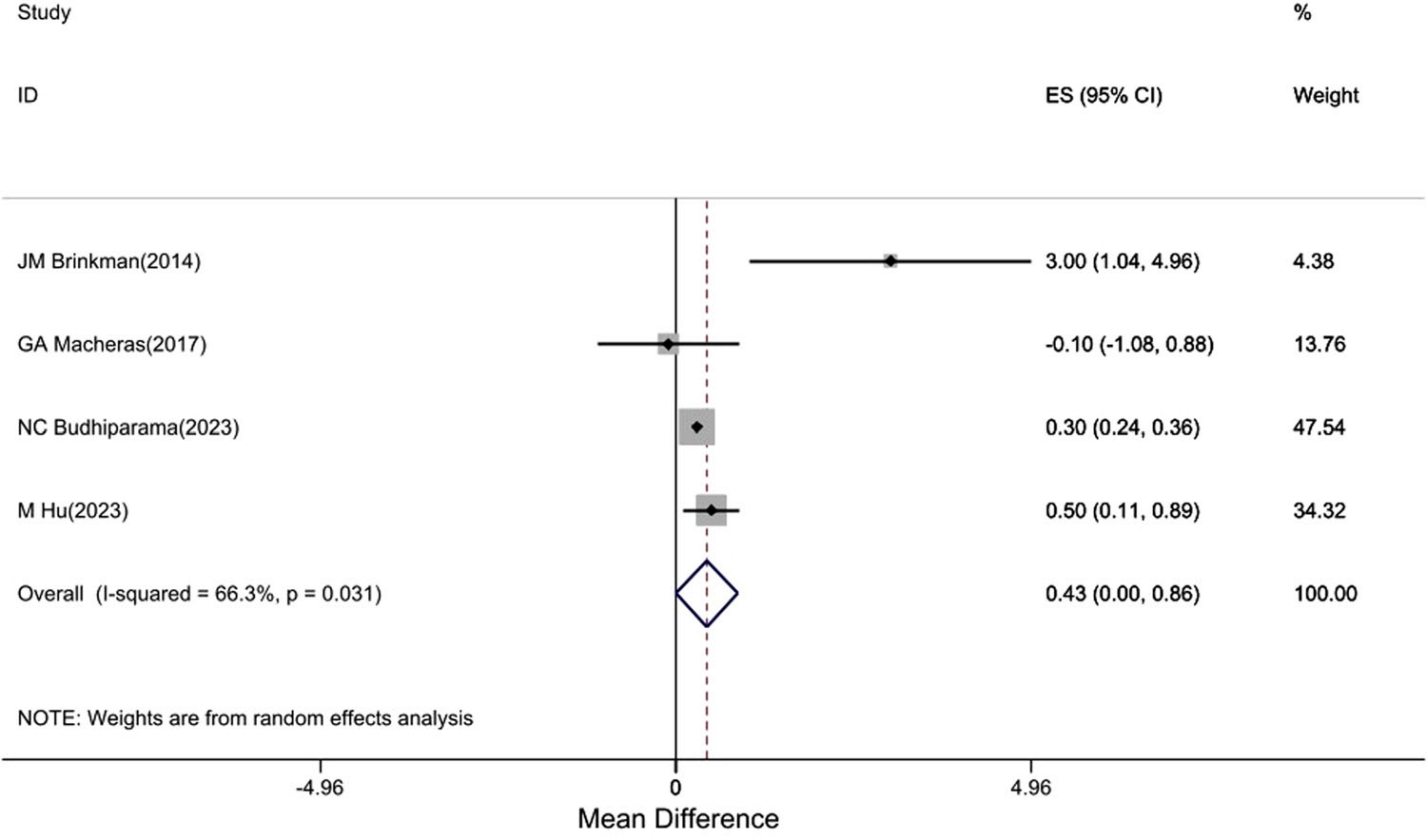
Difference in mean postoperative OKS score between CR and CS groups positive values favour CR). CI, confidence interval; CR, cruciate‐retaining; CS, cruciate‐sacrificing; ES, effect size; OKS, Oxford Knee Score. *Note*: Weights are from random effects analysis.

Four studies [8, 9, 16, 20] reported OKS with very high heterogeneity (*I* ^2^ = 89.6%) (Figure 5). Individual study results consistently showed differences below the MCID reference value of 4.5 points:

- Study differences ranged from −2.1 to +1.9 points.
- No individual study showed clinically meaningful differences.
- Prediction interval: −8.7 to 9.1 points.

#### Range of motion (ROM)

**Figure 6:**
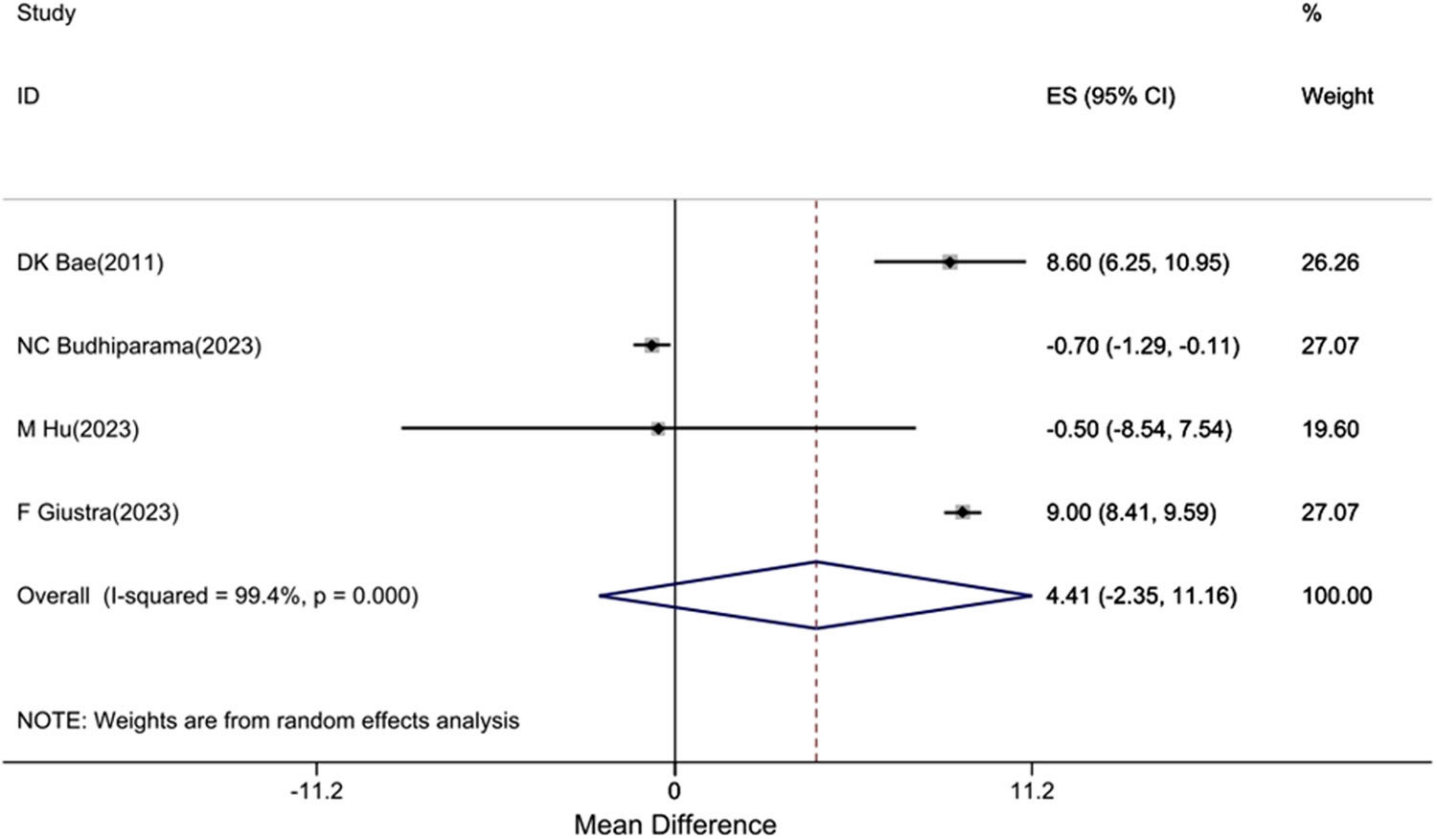
Difference in mean postoperative ROM score between CR and CS groups positive values favour CR). CI, confidence interval; CR, cruciate‐retaining; CS, cruciate‐sacrificing; ES, effect size; ROM, range of motion. *Note*: Weights are from random effects analysis.

Four studies [2, 9, 13, 16] reported ROM with extremely high heterogeneity (*I* ^2^ = 99.4%), making pooled analysis inappropriate (Figure 6). Individual study results varied widely, but none showed clinically significant differences (MCID reference value = 5.7°):

- Study differences ranged from −4.2° to +3.8°.
- High variability likely due to different measurement protocols and rehabilitation approaches.

### Complication analysis

**Figure 7:**
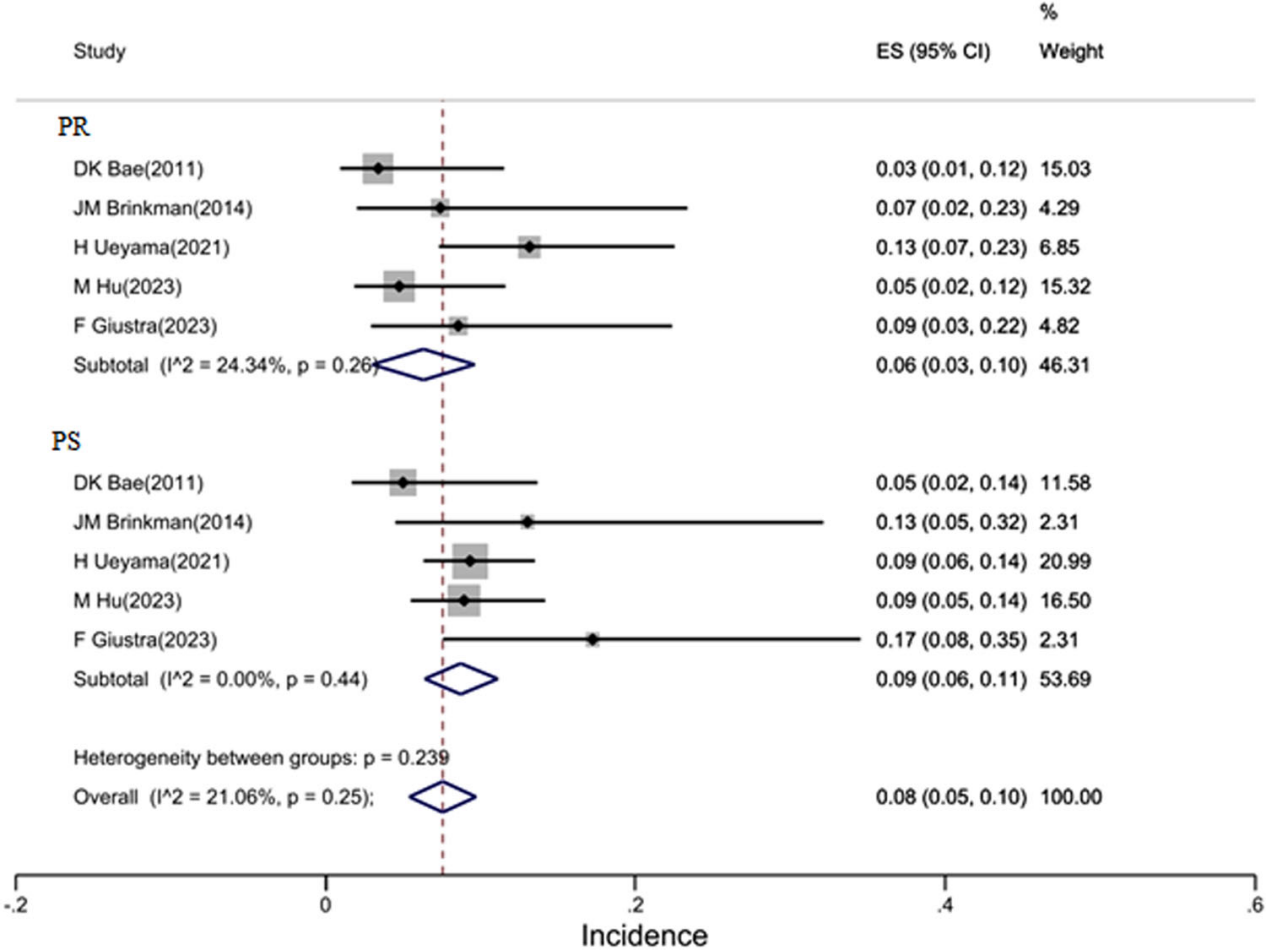
Overall incidence of postoperative complications and by group. CI, confidence interval; ES, effect size; PR, PCL retaining; PS, PCL sacrificing.

Five studies reported complications by group with low heterogeneity (*I* ^2^ = 21%), providing confidence in the pooled estimate. The overall complication rate was 8% (95% CI: 5%–10%). Subgroup analysis showed 9% complications in CS group versus 6% in CR group (*p* = 0.57), which was not statistically significant. Common complications included infection (2.1%), loosening (1.8%) and stiffness (1.5%) (Figure 7).

### Heterogeneity exploration

Meta‐regression identified several factors significantly associated with effect sizes (Table 2):

- Larger sample size associated with smaller effect sizes (*β* = 0.59, standard error [SE] = 0.18, *p* = 0.001).
- Older mean age associated with reduced effect sizes (*β* = −0.21, SE = 0.11, *p* = 0.031).
- Poor study quality associated with reduced effect sizes (*β* = −0.61, SE = 0.21, *p* = 0.011).
- Longer follow‐up associated with larger effect sizes (*β* = 0.15, SE = 0.08, *p* = 0.021).
- Asian studies associated with reduced effect sizes (*β* = −0.44, SE = 0.18, *p* = 0.009).

**Table 2:** Meta‐regression model of the effect of variables on effect size.

Variable	*β*	SE	*p*
Sample size	0.59	0.18	0.001
Mean age	−0.21	0.11	0.031
study quality (poor)	−0.61	0.21	0.011
Follow‐up	0.15	0.08	0.021
Geographical regions (Asian)	−0.44	0.18	0.009

Abbreviation: SE, standard error.

These findings suggest that study characteristics significantly influence outcomes, contributing to the observed heterogeneity.

### Sensitivity analysis

Sensitivity analyses revealed that studies with sample sizes <50 patients significantly influenced overall outcomes. When these smaller studies were excluded, effect estimates became more conservative and confidence intervals narrower. Studies with follow‐up <3 years also showed greater effect variability.

### Publication bias

**Figure 8:**
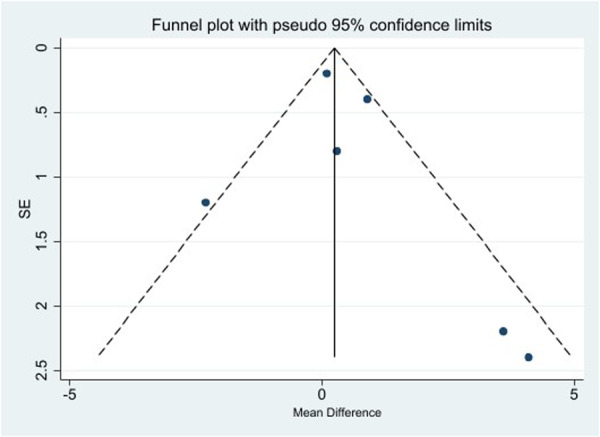
Bias publication assessment in the funnel plot.

With only seven studies, formal assessment of publication bias using Egger's test lacks adequate power. Visual inspection of available data suggested no obvious publication bias, but this assessment should be interpreted cautiously given the limited number of studies (Figure 8).

### GRADE evidence assessment

Using the GRADE approach, evidence certainty is low to moderate due to study design (all observational studies [no randomized controlled trials (RCTs)]—downgraded one level), heterogeneity (extremely high heterogeneity for key outcomes—downgraded one level), imprecision (wide confidence intervals for several outcomes—downgraded one level), geographic bias (predominance of Asian studies may limit generalizability—downgraded one level) and publication bias (cannot adequately assess with only seven studies).

## DISCUSSION

This systematic review found no clinically meaningful differences in functional outcomes or complication rates between PCL preservation and sacrifice in medial pivot TKA. However, it is crucial to emphasize that the certainty of evidence is significantly limited by extremely high heterogeneity (*I* ^2^ > 89%) for most functional outcomes, which severely limits the reliability of pooled results and prevents robust conclusions regarding the superiority of either approach.

The most important finding is that all observed differences were consistently below established MCID reference values across all functional outcome measures and individual studies. While MCIDs were originally developed to assess individual patient improvement rather than between‐group comparisons, the consistent pattern of small differences below these reference values suggests that any differences between approaches are unlikely to be clinically meaningful. This pattern suggests genuine clinical equipoise regarding PCL management in medial pivot TKA, supporting current practice variability where decisions are individualized.

The absence of clinically meaningful differences has important practical implications. PCL management decisions can be individualized based on surgical expertise and comfort with each technique, intraoperative assessment of PCL integrity and knee stability and technical considerations such as ease of soft tissue balancing. In addition, patients can be informed that PCL management strategy is unlikely to significantly impact their functional outcomes, allowing focus on other important factors. Finally, surgeons can develop expertise in either approach without concern about compromising patient outcomes.

The findings align with Verra et al.'s meta‐analysis of conventional prostheses, which found no clinically significant differences with PCL management [30]. Similarly, Movassaghi et al. reported equivalent outcomes regardless of PCL status in modern TKA [24]. However, this study specifically addresses medial pivot designs, which have unique kinematic properties that might theoretically alter the importance of PCL preservation.

The overall complication rate in this analysis (8%) is comparable to Cacciola et al.'s systematic review of medial pivot prostheses (6.6%) [10], supporting the reliability of these safety findings and confirming that PCL management strategy does not significantly influence safety outcomes.

Several important limitations affect the interpretation of these findings and must be acknowledged when considering the clinical implications of this systematic review. Most critically, the extremely high heterogeneity (*I* ^2 ^> 89% for most functional outcomes) severely limits confidence in pooled estimates and reflects substantial differences across multiple domains. This heterogeneity prevents definitive conclusions about the superiority of either approach and represents the most significant limitation of this analysis. The small number of included studies (*n* = 7) substantially limits statistical power and the ability to detect subtle differences or assess publication bias. Furthermore, all included studies were observational cohorts with no RCTs available, which introduces potential selection bias in PCL management decisions. Most studies did not adequately describe the decision‐making process for PCL preservation versus sacrifice, potentially introducing systematic differences between treatment groups. The extremely high heterogeneity (*I* ^2^ > 89% for most functional outcomes) severely limits confidence in pooled estimates and reflects substantial differences across multiple domains. This heterogeneity likely stems from several sources: technical factors (variations in PCL preservation techniques and soft tissue balancing approaches), study design variability (different patient selection criteria, surgical techniques and outcome measurement protocols), geographic factors (cultural and lifestyle differences affecting outcome expectations) and temporal factors (evolution of surgical techniques and perioperative care over the study period). The predominance of Asian studies (57%) may limit generalizability to other populations, particularly given the meta‐regression finding that geographic region significantly influenced effect sizes. This geographic bias restricts the external validity of these findings to more diverse patient populations. Additionally, there were insufficient data available on patient‐reported outcomes and satisfaction measures, limiting the ability to comprehensively evaluate the patient experience and subjective benefits of PCL preservation strategies. The heterogeneous study methodology, including variations in outcome measurement protocols, surgical techniques and follow‐up procedures, further compounds these data limitations.

Despite these limitations, this study has several notable strengths. This represents the first systematic review specifically examining PCL preservation in medial pivot prostheses, addressing an important gap in the literature. A comprehensive search strategy across multiple databases was employed, appropriate statistical methods that acknowledged heterogeneity through random‐effects modelling were used, and all limitations were transparently reported.

Given the limitations of current evidence, future research should prioritize well‐designed RCTs with standardized protocols and adequate power, consistent use of validated instruments with established MCIDs, advanced imaging to assess actual knee kinematics and stability, long‐term follow‐up to assess prosthesis survival and late complications beyond 10 years, and focus on satisfaction, quality of life and functional expectations.

In conclusion, based on current evidence with significant limitations, there are no clinically meaningful differences in functional outcomes or complication rates between PCL preservation and sacrifice in medial pivot TKA. However, the extremely high heterogeneity (*I* ^2 ^> 89%) for most functional outcomes severely limits the reliability of pooled results and prevents robust conclusions regarding the superiority of either approach. The consistent finding that all observed differences were below established MCID reference values across multiple studies and outcome measures suggests genuine clinical equipoise, though this conclusion must be interpreted cautiously given the substantial study heterogeneity. PCL management decisions should be individualized based on surgical expertise, patient anatomy and technical considerations rather than expected functional benefits. Given the low to moderate certainty of evidence and significant study heterogeneity, high‐quality RCTs with standardized protocols and adequate power are urgently needed to provide definitive guidance on PCL management in medial pivot TKA. Until such evidence becomes available, surgeons can make PCL management decisions based on individual expertise and patient factors without compromising functional outcomes.

## AUTHOR CONTRIBUTIONS

**Amir Mohsen Khorrami**: Design; data collection. **Mehdi Mohammadpour**: Data collection; analysis. **Mohammad Rastegar**: Data collection; writing—original draft. **Mohammad Ayati Firoozabadi**: Validation; writing—review and editing. **Seyed Mohammad Javad Mortazavi**: Conceptualization; supervision; writing—review and editing.

## CONFLICT OF INTEREST STATEMENT

The authors declare no conflicts of interest.

## ETHICS STATEMENT

The authors have nothing to report.

## Data Availability

Data supporting the findings of this study are available upon reasonable request from the corresponding author.
